# Smart Hydrophobic Surfaces: Nature-Inspired Designs for Sustainable Nanostructure Technologies

**DOI:** 10.3390/nano16130809

**Published:** 2026-06-30

**Authors:** Aigerim G. Zhaxybayeva, Muhammad Hashami, Meruyert Nazhipkyzy, Nakhypbek U. Aldiyarov, Saltanat S. Kaliyeva, Nazira B. Kassenova, Aina S. Khamitova, Altynbek A. Zhaparov, Adlet T. Otenov

**Affiliations:** 1 Department of Chemistry, Institute of Natural Sciences, L.N. Gumilyov Eurasian National University, Kazhymukan Str. 11, Astana 010008, Kazakhstan; zhaxybayeva_ag_1@enu.kz; 2 Department of Chemistry, Education Faculty, University of Mirwais Khan Nika Zabul, Qalat City 4001, Afghanistan; mg.hashami2010@gmail.com; 3 Department of Chemical Physics and Material Science, Faculty of Chemistry and Chemical Technology, Farabi University, Al-Farabi Ave 71, Almaty 050040, Kazakhstan; adlet240603@gmail.com; 4 Institute of Combustion Problem, Bogenbay Batyr Str. 178, Almaty 050012, Kazakhstan; 5 Department of Automation and Control, Satbayev University, Satbayev Str. 22, Almaty 050000, Kazakhstan; nahip16@mail.ru; 6 Higher School of Natural Sciences, Astana International University, Astana 010008, Kazakhstan; saltanat_kalieva@aiu.edu.kz; 7 Department of Chemistry and Biotechnology, Pedagogical Institute, Kokshetau University Named After Sh. Ualikhanov, Kokshetau 020000, Kazakhstan; nazira_kassenova09@mail.ru (N.B.K.); hamitova@mail.ru (A.S.K.); 8 Faculty of Mechanics and Mathematics, Farabi University, Al-Farabi Ave 71, Almaty 050040, Kazakhstan; altynbek.zhaparov@gmail.com

**Keywords:** superhydrophobicity, lotus effect, hydrophobic coatings, self-cleaning surfaces, hybrid nanomaterials

## Abstract

Hydrophobic and superhydrophobic surfaces have emerged as key solutions for fluid transport, biofouling prevention, and energy efficiency, with market forecasts projecting a compound annual growth rate (CAGR) of over 15% through 2030 due to their broad range of applications. This review critically examines the principles of natural hydrophobicity, as exemplified by lotus leaves and shark skin, and their translation into engineered surfaces via micro/nanofabrication techniques, such as laser patterning, etching, and self-assembly. Recent advances in hybrid nanomaterials have demonstrated WCAs in the range of 140–160°, along with enhanced mechanical strength and chemical stability, enabling applications in self-cleaning, anti-corrosion, and oil–water separation technologies. Superhydrophobic coatings are particularly important for reducing ice adhesion by more than 80%, while drag reduction in pipelines can reach up to 30%, contributing to energy savings. Despite these advances, challenges remain in achieving long-term stability under harsh environmental conditions, minimizing environmental impact, and developing cost-effective, scalable fabrication techniques. Future directions focus on environmentally friendly, multifunctional nanocomposites with switchable wettability, including pH- and light-responsive coatings capable of reversibly transitioning between superhydrophilic (<5°) and superhydrophobic (>150°) states, paving the way for sustainable and adaptable surface technologies.

## 1. Introduction

Hydrophobicity, defined as the tendency of a surface to repel water, has become a central design principle in the development of smart nanostructured materials owing to their wide-ranging applications in self-cleaning coatings, anti-fouling, anti-icing, and oil–water separation systems [1,2]. The growing industrial demand for hydrophobic coating is projected to exceed USD 3.5 billion by 2032, further highlighting the increasing demand across the energy, transportation, biomedical, and environmental remediation sectors [3].

**Figure 1:**
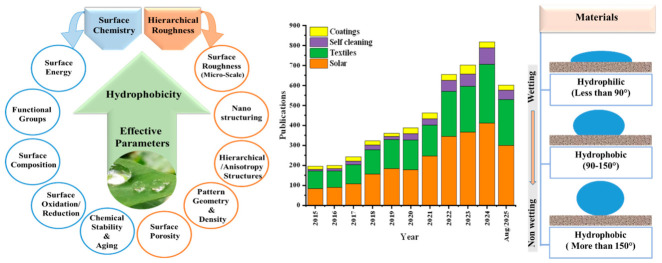
Transition from hydrophilic to superhydrophobic states based on contact angle measurements, highlighting the underlying scientific principles and key influencing parameters of hydrophobicity. The figure also illustrates the rapid expansion of research interest across major application areas from 2015 to 2025. Data were retrieved from the Scopus database using the search terms “Hydrophobic”, “Superhydrophobic”, and “Nanomaterials” within Applications.

Advances in large-scale, environmentally responsive, and mechanically robust hydrophobic systems are still piecemeal, and substantial gaps remain in translating the performance metrics found in the laboratory to the operational requirements in the field. While various studies focus on new fabrication techniques for the fabrication of hydrophobic and superhydrophobic materials, the evaluation of the performance in a standardized manner under appropriate environmental conditions posing suitable stress factors is still an unsolved issue. This leads to the urgent need for a critical, integrative review of the material and structural innovations of a state-of-the-art that not only gathers the material and structural novelties but also weighs them on the scales of scalability, sustainability, and multifunctional reliability. In the context of this review, smart hydrophobic surfaces refer to engineered systems that exhibit adaptive, stimuli-responsive, self-healing, multifunctional, or controllable wetting behavior beyond conventional passive water repellency. Such surfaces can dynamically respond to environmental triggers, including light, temperature, pH, electric fields, mechanical damage, or chemical stimuli, enabling tunable wettability and enhanced functionality for advanced technological applications. Surface parameters controlling hydrophobicity and the rising research interest from (2015 to 2025) in diverse applications, such as coatings, textiles, and solar devices, are presented in Figure 1.

The most popular example of naturally occurring superhydrophobic materials is the lotus effect, which was systematically studied in the 1990s. The leaves of lotus are characterized by dual-scale surface morphology, in which papillae of micrometres are topped by nanoscale wax crystals that entrap air and dramatically decrease liquid–solid contact [4]. This micro/nanostructure facilitates the Cassie–Baxter wetting state, where droplets of water roll off, and contaminants are removed, thereby giving the micro/nanostructure a self–cleaning capability [5]. In addition to lotus leaves, similar wetting mechanisms are found in rose petals, rice leaves, butterfly wings, and cicada wings, each adapted to fulfil specific biological functions [6,7]. These naturally optimized designs have inspired biomimetic material engineering, where the translation of such principles into synthetic architecture not only replicates but often surpasses natural performance metrics.

Building on this biological inspiration, recent literature highlights a rapid and multidirectional progression in the fabrication and functionalization of engineered hydrophobic and superhydrophobic surfaces. Advances in scalable coating techniques, such as spray-coating, chemical vapor deposition, soft lithography, and integrated top-down/bottom-up structuring, have enabled the reproducible fabrication of hierarchical architectures that routinely exhibit water contact angles above 150° and sliding angles below 10° [8]. Increasing emphasis is also placed on mechanical durability, with coatings retaining over 90% of their repellency after repeated abrasion or tape-peeling cycles, and demonstrating long-term stability under UV exposure or chemical immersion for hundreds of hours [8,9].

Anti-icing and de–icing functionalities have only recently emerged as an active research focus. The integration of engineered micro/nanostructures with low-surface-energy chemistries has been shown to reduce ice adhesion by up to 90% compared with untreated substrates while extending ice accretion delay times by several minutes even at temperatures as low as (−20 °C) [9]. Furthermore, hierarchical dandelion-like morphologies incorporating photothermal nanostructures can harness solar energy to induce localized heating, enabling passive and energy-efficient de-icing, with reported ice–removal time more than 60% shorter than those of non–photothermal counterparts [10].

Hydrophobic surface modification strategies span from traditional approaches such as wax coatings, fluorinated polymers, and silanization to advanced engineered systems based on nanostructured coatings, patterned lithography, plasma texturing, electrospinning, and layer–by–layer self–assembly [11]. Increasingly, sustainability goals are driving the evolution of these technologies. For example, lignin–reinforced rubber films combine high oil–water separation efficiency (>98%) with mechanical flexibility and recyclability [12]. At the same time, magnetic soot-based sorbents demonstrate both high oil uptake (>40 g g^−1^) and rapid magnetic recovery for spill remediation [13]. Naturally derived hydrophobic materials, including cellulose nanofibers, are being incorporated into coating matrices as substitutes for fluorinated chemicals, thereby lowering environmental impact without compromising repellency [3,14].

Bioinspired strategies are increasingly diversifying. Recent advances in re–entrant geometries and hierarchical multiscale designs have enabled superomniphobic surfaces capable of repelling not only water but also low–surface–tension liquids, such as ethanol and hexadecane, thereby extending applications from microfluidics to hazardous chemical containment [12,15,16]. The high-dimensional design space defined by surface morphology, chemistry, and durability is now being explored computationally, with improved lotus–effect optimization algorithms accelerating both material discovery and performance prediction [17,18].

Biomedical applications are rapidly advancing. Hydrophobic micro/nanostructured polymer–nanoclay composites have demonstrated low protein adsorption, high hemocompatibility, and enhanced endothelialization, making them promising candidates for cardiovascular stents and catheters [19]. Glass–based substrates with tailored wetting properties are also enabling progress in diagnostics, biosensing, and tissue engineering [20]. Such multifunctional capabilities, including anti–fouling, anti–icing, corrosion resistance, and photothermal or antimicrobial activity, mark a clear transition from proof-of-concept demonstrations to application–ready technologies [8,14].

Current developments include superomniphobic surfaces that repel a wide range of liquids beyond water, expanding potential applications in chemical protection, hazardous liquid handling, and biofluid management [16]. However, progress remains fragmented across different fields, with researchers and industry alike struggling to identify the most effective combinations of materials, fabrication processes, and functionalities for specific applications. An integrative framework that links fundamental theory, bioinspired design principles, scalable production methods, and multienvironment performance testing will be essential to bridge the gap between academic exploration and commercial implementation. The convergence of biological inspiration, advanced fabrication, sustainability, and multifunctionality positions engineered hydrophobicity as a transformative platform across diverse domains, including energy, environmental remediation, healthcare, and aerospace.

While previous reviews have primarily focused on individual aspects of hydrophobic surface engineering, such as wetting mechanisms, fabrication methods, biomimetic designs, or specific applications, this review presents a unified framework linking wetting theory, bioinspired design principles, material innovations, fabrication technologies, durability assessment, sustainability considerations, and application–oriented performance evaluation. The progression from naturally derived hydrophobic materials to advanced synthetic architectures is then examined, with emphasis on fabrication techniques, material selection, and structural design strategies. By correlating surface chemistry, hierarchical structuring, and fabrication methods with application-specific performance, this review provides a unified perspective that bridges the gap between fundamental wetting theory and industrial deployment. Special emphasis is placed on emerging multifunctional and stimuli–responsive systems, highlighting how adaptive wettability and environmentally benign materials can redefine next–generation hydrophobic technologies. This integrative approach distinguishes the present review from existing literature and offers a structured roadmap for the rational design and evaluation of sustainable hydrophobic surfaces.

## 2. Fundamentals of Hydrophobicity

**Figure 2:**
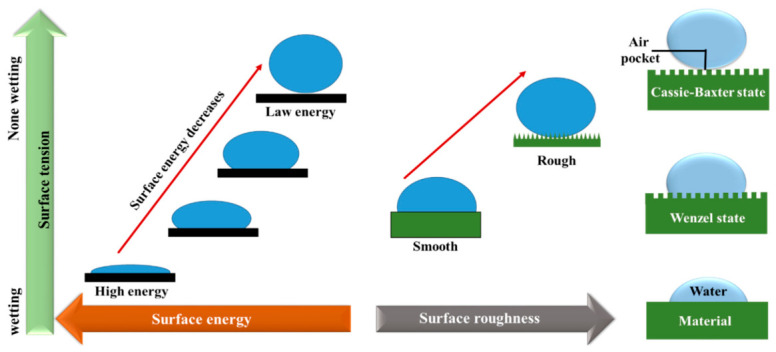
Schematic illustration of hydrophilic, hydrophobic, and superhydrophobic surfaces, showing their relation to surface free energy and surface roughness, and highlighting how nanoscale texturing combined with low–energy surfaces drives the transition from hydrophilic to superhydrophobic states.

Hydrophobicity refers to the repulsion of water on the surface due to low surface free energy, representing a fundamental concept in surface science and interfacial engineering [21]. Its extreme form, superhydrophobicity, is defined by water contact angles exceeding 150° and sliding angles typically below 10°, allowing water droplets to roll off with minimal adhesion [22]. These properties arise from the combination of surface chemistry and hierarchical micro/nanostructuring, which control the wetting regime as described by the Wenzel and Cassie–Baxter models [11]. The degree of hydrophobicity is typically quantified by the water contact angle (CA), which reflects the interfacial interactions at the liquid–solid–vapor boundary [23,24]. Surfaces with contact angles below 90° are considered hydrophilic, whereas those above 90° are hydrophobic. When the contact angle exceeds 150°, and the sliding angle falls below 10°, the surface is described as superhydrophobic, enabling water droplets to roll off with ease [25,26]. Such extreme wetting states are technologically significant, as they underpin the lotus effect and serve as design paradigms for next–generation nanoengineered surfaces. Figure 2 schematically illustrates these distinct wetting regimes, hydrophilic, hydrophobic, and superhydrophobic, through their characteristic contact angles, providing a clear conceptual framework for understanding the progression toward extreme water repellency.

Young’s Equation (1) provides the theoretical basis for wetting, describing the equilibrium contact angle on an ideal smooth and chemically homogeneous surface, determined by the balance of solid–vapor (SV), solid–liquid (SL), and liquid–vapor (LV) interfacial energies [27]. In practice, however, surfaces deviate from this idealized picture due to inherent roughness and chemical heterogeneity. The Wenzel model extends Young’s framework by introducing a roughness factor (*r*), defined as the ratio of actual to projected surface area (*r* > 1) (2). This model shows that surface roughness amplifies the intrinsic wettability: hydrophilic surfaces become more hydrophilic, whereas hydrophobic surfaces become more hydrophobic. The balance between the dispersive and polar components of surface free energy also strongly influences hydrophobicity. An increase in polar surface groups due to oxidation, ultraviolet exposure, or chemical aging enhances water affinity and gradually reduces water repellency. Therefore, maintaining long-term hydrophobic performance requires preserving both the hierarchical surface structure and stable surface chemistry.

In contrast, the Cassie–Baxter model (3) describes wetting on heterogeneous or textured surfaces, where air pockets become trapped beneath the liquid droplet. This reduces the effective solid–liquid contact area, weakens adhesion, and stabilizes large apparent contact angles. In this model, *f*_s_ represents the fraction of the solid–liquid interface and *f*_v_ the fraction of the liquid–vapor (air) interface [26]. In nanotechnology-driven hydrophobic surfaces, the Cassie–Baxter framework provides the theoretical basis for how nanoscale patterning and hierarchical roughness enable the transition to superhydrophobicity. A classic example is the lotus leaf, which exhibits contact angles of 160–170° and sliding angles as low as 2–5°, inspiring a wide range of biomimetic nanofabrication strategies [25].

(1) $$Cos\;\theta_{y}=\frac{YSV-YSL}{YLV}$$

(2) $$Cos\;\theta_{w}=r\;Cos\;\theta_{y}$$

(3) Cos θCB=fsCos θy+fvCos θv 

Surface energy and surface roughness are the two primary parameters used to tailor hydrophobicity. Hydrophobic materials typically exhibit low surface free energies (e.g., fluoropolymers, 10–20 mN/m), whereas hydrophilic materials are characterized by higher surface energies (>500 mN/m) [28]. Roughness plays a particularly critical role in nanostructured systems, where designed surface asperities can drastically modify wetting behavior. For instance, roughened glass beads have been shown to alter flotation characteristics by changing the apparent contact angle, underscoring the influence of topography on surface–fluid interactions [29]. Similarly, the hydrophobic stability of oxide layers, such as Er_2_O_3,_ has been demonstrated to depend more on surface geometry than on surface chemistry, highlighting the synergistic role of nanoscale texturing in maintaining performance under adverse environmental conditions [30].

To clearly and graphically illustrate how physicochemical and structural parameters affect wettability, the role of each factor in controlling hydrophobic and superhydrophobic behavior is summarized in Table 1. Surface energy, chemistry, roughness, morphology, and hierarchical structuring each play a distinct role in determining the apparent contact angle and the stability of wetting states. For example, fluoropolymers, with intrinsically low surface free energies (10–20 mN/m), exhibit contact angles above 100°, whereas unmodified high–energy materials remain strongly hydrophilic. Similarly, nanoscale roughening enhances the intrinsic wetting behavior, with roughened glass beads showing a contact angle shift from ~65° to >95° [29], while lotus-inspired hierarchical structures achieve extreme repellency, with contact angles above 160° and sliding angles as low as 2–5° [25,26]. These comparisons emphasize that hydrophobicity is not governed by a single parameter but by the synergy of surface chemistry and multiscale topography, which together determine the durability and functionality of smart nanostructured surfaces.

Advances in smart nanotechnologies have demonstrated that durable hydrophobicity requires the co–engineering of both surface chemistry and topography. On the chemical side, low–energy functional groups (e.g., CF_3_, CH_3_) and siloxane linkages reduce water affinity, while on the structural side, micro/nanoscale texturing enables composite wetting states by minimizing the solid–liquid contact area [31]. This dual control provides opportunities for designing adaptive surfaces capable of switching wettability, resisting biofouling, and maintaining performance under dynamic environments. More recently, nanotexturing and plasma-based techniques have enabled the fabrication of anti-reflective, highly hydrophobic hierarchical surfaces with contact angles exceeding 160°, while simultaneously minimizing optical losses, an essential requirement for optical coatings and solar cell applications [33].

A unifying feature of state-of-the-art superhydrophobic technologies is the integration of hierarchical structuring across multiple length scales. In nature, lotus leaves and insect wings achieve their extreme water repellency through microscale protrusions decorated with nanoscale features, which stabilize the Cassie–Baxter state. Replication of such multiscale architectures has been achieved using scalable approaches, such as nanoimprint lithography, plasma etching, and self–assembly, yielding non-wetting surfaces that retain high water repellency even under abrasion or contamination [32]. Importantly, hierarchical structuring also enhances durability. The microscale framework can maintain hydrophobic functionality even when nanoscale features are partially degraded, a property critical for long-term performance in coatings, textiles, and biomedical devices [30].

Hydrophobicity and superhydrophobicity are not merely surface phenomena but have become guiding principles in the design of smart nanotechnologies. Their theoretical foundation is provided by classical wetting models, such as Young, Wenzel, and Cassie–Baxter, which are increasingly being translated into practical technologies through nanoscale fabrication and hierarchical patterning. The interplay of tailored surface chemistry, energy minimization, and multiscale roughness enables the creation of surfaces with unprecedented repellency, self–cleaning capability, and stability. Looking forward, the future of hydrophobic smart technologies lies in the integration of nano–engineered chemistries with hierarchical architectures, paving the way for multifunctional, durable, and adaptive surfaces for applications in energy, environmental remediation, and biomedicine.

### 2.1. Lotus Effect

**Figure 3:**
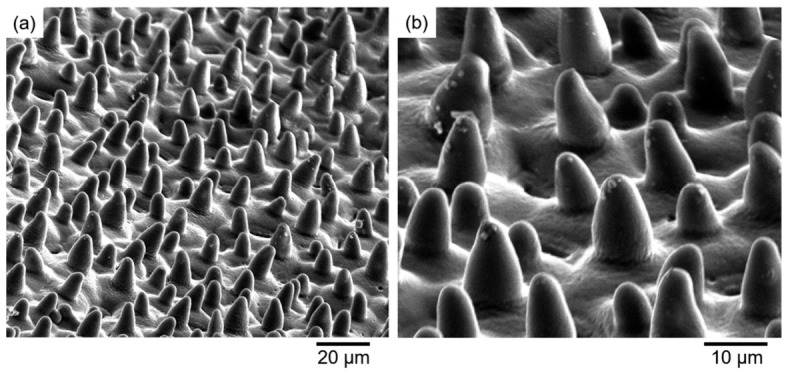
SEM images of the natural lotus leaf surface at different magnifications: (**a**) low-magnification view and (**b**) high-magnification view. Reprinted from Wang et al. (2021) [36] under the CC BY 4.0 license.

Bioinspiration has become an influential design paradigm, with natural surfaces serving as functional templates for the development of advanced materials. Among the most studied examples is the lotus effect, a striking manifestation of superhydrophobicity in which water droplets roll off the surface, removing dirt and contaminants in the process. Engineered surfaces that mimic this effect commonly exhibit water contact angles above 150° and sliding angles below 10°, demonstrating their utility in self-cleaning, anti-fouling, and protective coatings [34,35]. The surface morphology of lotus leaves plays a central role in their hydrophobicity. Figure 3a,b show the characteristic surface morphology of a natural lotus leaf, consisting of densely distributed microscale papillae with a predominantly conical geometry. Based on the scale bars, the protrusions exhibit dimensions on the order of approximately 10–20 μm and are uniformly distributed across the leaf surface. The higher–magnification image in Figure 3b further reveals the pronounced three-dimensional nature of these structures, which contribute to a highly rough surface topography. These microstructured architectures are widely recognized as key factors governing the hydrophobic behavior of lotus leaves by reducing the effective solid–liquid contact area and facilitating air entrapment beneath water droplets [36]. Such morphologies improve self–cleaning and anti–fouling performance, demonstrating the successful transfer of bioinspired concepts to engineered substrates [36,37].

Artificial surfaces mimicking these features have qualitatively replicated the natural lotus repellency, with contact angles exceeding 160° and excellent wetting durability [38]. Beyond the lotus leaf, other natural systems inspire the design of hydrophobic and adhesive surfaces. Rose petals, for example, exhibit the so–called “rose petal effect,” where water droplets display high contact angles (>150°) yet remain pinned to the surface. This is caused by nanoscale cuticular folds superimposed on micropapillae, creating strong droplet adhesion. Such properties are valuable for applications requiring droplet immobilization, such as microfluidics and controlled biochemical assays [39].

Other systems reveal alternative design strategies. Butterfly wings integrate optical and wetting mechanisms, in which overlapping scales not only enhance iridescence but also generate anisotropic hydrophobicity, which guides droplets in preferred directions. Water striders use dense arrays of superhydrophobic leg hairs to exploit surface tension, enabling them to walk on water. Together, these examples highlight that natural hydrophobicity is multifunctional, ranging from self–cleaning and droplet immobilization to directional transport and flotation [40]. The wetting mechanisms and structural strategies of these natural models can be systematically compared to highlight their functional diversity and technological significance. As summarized in Table 2, lotus leaves, rose petals, butterfly wings, and water striders each exploit distinct hierarchical morphologies and surface chemistries to achieve superhydrophobicity, droplet pinning, anisotropic rolling, and buoyancy, respectively.

In biomedical contexts, hydrophobic biomimetic surfaces are applied to implants, catheters, and biosensors to reduce bacterial adhesion and biofouling, thereby improving both device longevity and biocompatibility [42,43].

Nature provides strong conceptual models; however, in the context of artificial reproduction, there is often a trade–off between structural integrity, scalability, and durability. The diversity of natural strategies from rose petals to water striders demonstrates that nature’s solutions are varied and multifunctional.

### 2.2. Hydrophobic Nanomaterials

**Figure 4:**
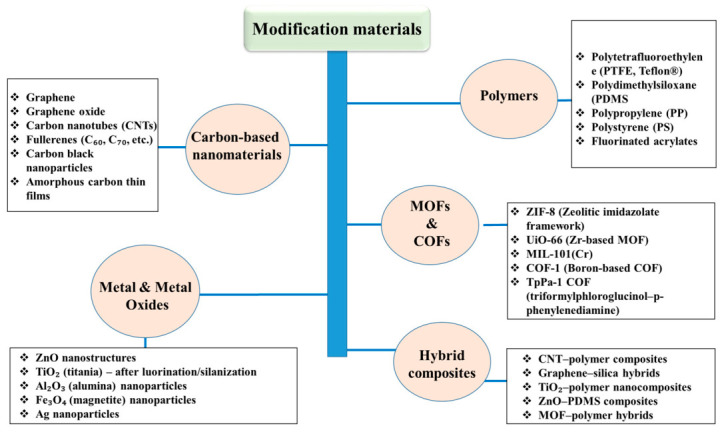
Modification materials for hydrophobic coatings, showing carbon–based, metal/metal oxide, polymeric, MOF/COF, and hybrid systems with their associated functionalities.

Among the most established classes, carbon-based nanomaterials (CBMs), including carbon nanotubes (CNTs), graphene, and carbon nanofibers, exhibit intrinsic hydrophobicity, large aspect ratios, and chemical stability. Figure 4 schematically categorizes the major classes of modification materials, including carbon-based nanomaterials, metals and metal oxides, polymers, MOFs/COFs, and hybrid composites, highlighting their versatility in enabling scalable, multifunctional, and durable hydrophobic coatings. Polymers provide robust coatings with excellent hydrophobicity and dirt/allergen repellency, while their inherent porosity facilitates air entrapment at the solid–liquid interface [44,45]. Metal and metal oxide nanomaterials, such as copper oxide, zinc oxide, and silica, are widely employed due to their ease of synthesis, hierarchical porosity, and compatibility with surface functionalization strategies. These materials enable low-cost, scalable coatings with tunable hydrophobicity, and recent studies have demonstrated their effectiveness in corrosion resistance and antifouling applications [46,47].

Polymeric nanomaterials provide flexibility in tailoring surface chemistry; in particular, fluorinated or silane-modified matrices exhibit excellent water repellency combined with mechanical stability. Metal–organic frameworks (MOFs) and covalent organic frameworks (COFs) are also promising candidates for selective wetting control owing to their intrinsic porosity, crystalline architecture, and highly tunable surface functionality. Their structural diversity suggests the potential for hybrid hydrophobic coatings with innovative properties, such as enhanced chemical resistance and reusability. Hybrid and composite nanomaterials, which integrate carbon-based, metal oxide, and polymeric components, represent a synergistic pathway toward hydrophobicity. For example, embedding CNTs within a fluoropolymer matrix in combination with silica nanoparticles has been shown to simultaneously enhance mechanical strength and increase the water contact angle, yielding a robust superhydrophobic surface [48]. Collectively, these material classes provide a versatile platform for the rational design of hydrophobic and superhydrophobic surfaces, paving the way for next–generation technologies in self–cleaning, anti–corrosion, and liquid separation.

Carbon-based nanomaterials, including carbon nanotubes (CNTs), graphene (G), and graphene oxide (GO), are regarded as universal building blocks for fabricating hydrophobic and superhydrophobic surfaces due to their tunable surface chemistry, ultrahigh aspect ratios, and inherent nanostructured roughness [49]. Carbon nanotubes have been widely employed in superhydrophobic coatings, where their intrinsic hydrophobicity, large surface area, and hierarchical topography enable the design of durable repellency. When combined with fluorinated polymers, CNT–based coatings have achieved water contact angles (WCAs) exceeding 165°, demonstrating strong superhydrophobicity together with mechanical robustness under abrasion [48]. Graphene and graphene oxide provide versatile two–dimensional platforms for surface functionalization. While pristine graphene shows only moderate hydrophobicity with WCAs of ~90–95°, chemical modification (e.g., fluorination or alkyl–silane treatment) can enhance its performance, achieving WCAs greater than 150° [46].

Surface modification is very important for enhancing the hydrophobicity of these nanomaterials. Functionalization with low–surface–energy moieties (such as –CF_3_ or –CH_3_ groups) decreases the surface free energy and increases water repellency [50]. Fluorosilane–modified GO surfaces showed WCAs of 155° with sliding angles less than 10°, suitable for self–cleaning and oil–water separation applications [44,51]. The nanoscale surface roughness produced by CNT bundles or graphene nanosheets also enhances the intrinsic hydrophobicity by allowing the Cassie–Baxter wetting regime to be achieved, in which air pockets are trapped underneath the water droplets [45]. The hydrophobic properties of CNTs, graphene, and GO under various modification strategies are summarized in Table 3, which shows the inherent wettability, tunability via surface engineering, and targeted functionalities of these materials.

In addition, carbon-based nanomaterials impart both chemical stability and multifunctionality. For example, sprayed CNT–silica–polymer hybrid coatings combine superhydrophobicity with high thermal resistance, while graphene-based surfaces exhibit excellent stability under corrosive and oxidative conditions [47]. Carbon-based nanomaterials, such as CNTs, graphene, and GO, provide a tunable spectrum of hydrophobic performance, ranging from moderate water contact angles (~90°) to extreme superhydrophobicity (>165) [52,53].

Graphene-based and CNT-enhanced films serve as corrosion barriers for metals in harsh environments, protecting against corrosion and chemical degradation [47]. In oil–water separation, functionalized GO or CNT membranes exhibit hydrophobic properties, selectively repelling water while capturing oils, with separation efficiencies exceeding 95% in laboratory studies [46]. Hybrid nanomaterials, such as CNT–silica–polymer composites, combine extreme hydrophobicity with mechanical robustness, thermal stability, and chemical resistance, underscoring the multifunctional potential of engineered surfaces [48].

**Figure 5:**
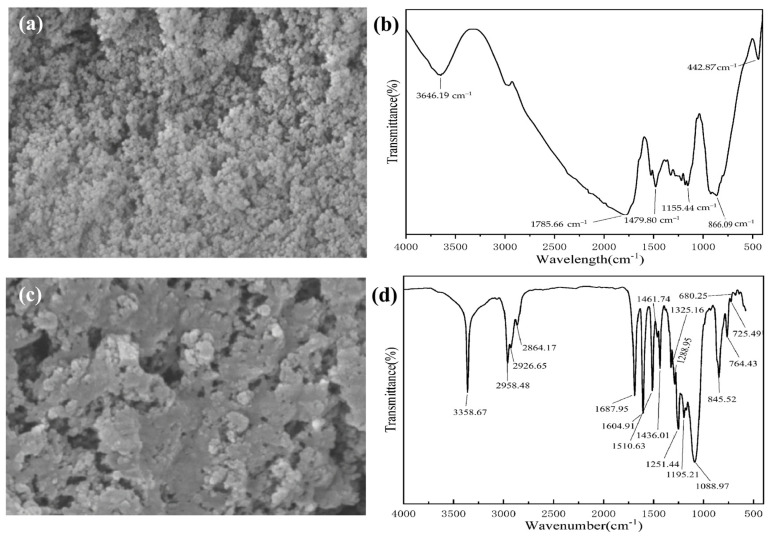
SEM and FTIR analyses of SiO_2_ and TiO_2_ coatings, showing surface chemistry and morphology differences between superhydrophilic (**a**,**b**) and superhydrophobic (**c**,**d**) states. [Adapted from Lu and Zheng (2022) [58], Coatings, 12(4), 502, under CC-BY 4.0].

Metal and metal oxide nanomaterials, such as ZnO, TiO_2_, SiO_2_, Al_2_O_3_, and Fe_3_O_4_, are widely used for engineering hydrophobic and superhydrophobic surfaces due to their tunable surface chemistry, high surface area, and mechanical stability [54,55]. While metal oxides are generally moderately hydrophilic or weakly hydrophobic, with WCAs ranging from 40° to 90° depending on particle size, morphology, and surface hydroxyl density, functionalization with low–surface–energy molecules, such as fluorosilanes, perfluoroalkyl chains, or long–chain alkyl groups, enables the formation of hydrophobic or superhydrophobic surfaces with WCAs up to 160° and sliding angles below 10° [56,57]. The SEM images and FTIR analyses in Figure 5a–d reveal the distinct morphological and surface chemistry differences between superhydrophilic and superhydrophobic coatings of SiO_2_ and TiO_2_.

Superhydrophilic films in Figure 5a display densely packed nanoparticles with high surface roughness and hierarchical porosity, promoting strong water spreading and rapid droplet absorption. Corresponding FTIR analysis in Figure 5b indicates the presence of surface hydroxyl groups at ~3690 cm^−1^ and Si–O/Ti–O stretching vibrations near 1100 cm^−1^, confirming hydroxyl-rich surfaces responsible for water affinity [58,59]. In contrast, superhydrophobic coatings in Figure 5c exhibit aggregated micro/nanostructures with air-trapping voids, while FTIR spectra in Figure 5d show reduced hydroxyl intensity together with additional peaks from hydrophobic functional groups, such as Si–O–Si and C–H stretching (2920–2960 cm^−1^). These modifications yield water contact angles exceeding 150° with sliding angles below 10°, consistent with engineered surfaces designed for self-cleaning, antifouling, and oil–water separation applications [60,61].

In the case of metal oxides, SiO_2_ nanoparticles are among the most widely used functionalized materials for superhydrophobic coatings. When fluorosilane-modified SiO_2_ incorporated into polymer matrices or electrospun membranes, they provide efficient self-cleaning and water–oil separation [62,63]. Similarly, TiO_2_ and ZnO nanoparticles, when functionalized with low-surface-energy molecules, act as both hydrophobes and photocatalysts, with applications in antifouling and environmental remediation [64,65]. Fe_3_O_4_ nanoparticles, due to their magnetic properties, represent multifunctional materials in which superhydrophobicity and magnetic separability are combined, a feature particularly useful for oil–water separation in environmental remediation [66]. Al_2_O_3_ nanoparticles have been shown to enhance hydrophobicity and the mechanical barrier properties of epoxy or polymer composites when added as fillers, achieving WCAs of 150–155° [55]. The hydrophobic performance and applications of these metal and metal oxide nanomaterials under surface functionalization are summarized in Table 4, emphasizing both WCAs and multifunctionality.

Superhydrophobic surfaces based on these coatings exhibit strong resistance to corrosion, wear, and chemical erosion, significantly enhancing surface lifetimes in industrial, marine, and environmental applications [56,67]. Water contact angles of 150–160° and low sliding angles highlight the effectiveness of surface engineering, while their versatility, scalability, and integration with additional functionalities, such as photocatalysis, magnetism, and mechanical reinforcement, demonstrate the crucial role of metal and metal oxide nanomaterials in hydrophobic surface design [54,57].

Polymeric nanomaterials, such as fluorinated polymers, polydimethylsiloxane (PDMS), and Teflon-like coatings, are widely used to prepare hydrophobic and superhydrophobic surfaces due to their inherently low surface energy and tunable chemical functionality [68,69]. Fluorinated polymers, in particular, dramatically reduce surface energy; for thin films, WCAs range from 120–140°, while hierarchical micro/nanostructured surfaces can achieve WCAs exceeding 160°. PDMS-based coatings are flexible and chemically stable, with WCAs between 110–150° depending on surface roughness and crosslinking density, making them suitable for self-cleaning and antifouling applications [70,71]. The high fluorine content of Teflon-like coatings results in WCAs above 150° and low sliding angles, ensuring strong hydrophobicity even under mechanical stress or chemical attack [68].

Nanocomposite polymer coatings where polymer matrices are made significantly stronger by reinforcing nanoparticles, such as SiO_2_, TiO_2_ or ZnO, providing even better surface roughness and durability for superhydrophobicity and mechanical robustness [72,73]. The combination of low-surface-energy polymers and nanoscale topography enables water droplets to adopt the Cassie–Baxter state, which decreases the solid–liquid contact and offers sliding angles below 10° [74,75]. Such surfaces are especially beneficial in medical devices, where low adhesion minimizes biofouling and in industrial pipelines, where water repellency improves flow efficiency [69,70].

The WCAs of polymeric nanomaterials can be tuned from moderate hydrophobicity (~110°) to extreme superhydrophobicity (>160°) depending on surface functionalization and morphology. Owing to their chemical stability, elasticity, transparency, and scalability, these materials find applications in self-cleaning surfaces, anti-corrosion coatings, biomedical devices, and water–oil separation technologies. Their tunability, low-cost processing, and incorporation into nanocomposite matrices underscore the important role of polymeric nanomaterials in engineered hydrophobic and superhydrophobic surface technologies [68,73]. A functional overview of polymeric nanomaterials is summarized in Table 5, highlighting strategies for achieving hydrophobic performance and targeted applications through various polymer and surface/nanocomposite approaches.

Metal–organic frameworks (MOFs) and covalent organic frameworks (COFs) are highly porous crystalline materials that have attracted significant attention for engineering hydrophobic, hydrophilic, and superhydrophobic surfaces, due to their tunable porosity, adjustable surface chemistry, and large surface area [76,77]. MOFs, including HKUST-1 and ZIF derivatives, provide inherently nanoporous structures that can become hydrophobic and enable oil–water separation when functionalized with hydrophobic groups or fluorinated ligands. Functionalized MOFs can achieve WCAs greater than 150°, while retaining porosity for selective adsorption and transport of target molecules [78,79].

COFs, assembled from organic monomers via covalent bonds, offer a complementary platform with highly tunable pore chemistry. Through surface engineering, such as fluorination, alkylation, or hybridization with MOFs, COFs can reach superhydrophobicity with WCAs exceeding 155°, accompanied by chemical and thermal stability [80,81]. Their high porosity reduces the solid–liquid contact area, favoring the Cassie–Baxter wetting regime and enhancing droplet mobility. These properties make COFs ideal candidates for hydrophobic coatings on textiles, chemically resistant films, and filtration membranes [77,82].

Hybrid MOF/COF materials leverage the complementary features of the two frameworks: MOF cores provide selective adsorption and structural stability, while COF shells confer hydrophobicity and mechanical strength. These composites have demonstrated water contact angles exceeding 155°, oil–water separation efficiencies above 95%, and have been successfully incorporated into devices such as humidity-independent olfactory sensors for plant disease diagnosis [79,83]. The functionalization methods, hydrophobicity, porosity, and performance of MOFs, COFs, and their hybrid composites are summarized in Table 6. The table illustrates how porosity and surface functionalization interact to achieve superhydrophobicity, with water contact angles in the range of 150–160°. MOFs are characterized by high surface area and selective adsorption capabilities, COFs by chemical and thermal stability with tunable pore chemistry, and MOF/COF composites integrate both sets of properties for enhanced performance. These materials are useful for oil–water separation, hydrophobic coatings, filtration membranes, and multifunctional sensing devices, clearly demonstrating their versatility in advanced hydrophobic surface engineering.

Porosity and surface functionalization are the basis of the importance of MOFs and COFs in the creation of advanced hydrophobic and multifunctional surfaces [76,85]. Hybrid and composite nanomaterials, which combine two or more materials, have demonstrated synergistic effects in the engineering of hydrophobic and superhydrophobic surfaces. Hybrid systems that integrate carbon-based nanomaterials with metal/metal oxide nanoparticles, polymers, or MOFs/COFs can synergistically tune surface roughness, reduce surface energy, enhance mechanical robustness, and provide multifunctionality [86,87]. For instance, incorporation of inorganic nanoparticles into polymer matrices or carbon-based frameworks can increase WCAs from moderate hydrophobicity (~100°) to superhydrophobicity (>160°), while maintaining structural integrity under mechanical stress or chemical exposure [88,89].

The hierarchical multiscale surface morphology can serve as an effective trapping mechanism in nanocomposite coatings, capturing air pockets that promote the Cassie–Baxter wetting regime and reduce droplet adhesion [90,91]. Magnetic nanoparticle–polymer composites combine magnetic responsiveness with superhydrophobicity, offering potential applications in environmental remediation, self-cleaning surfaces, and controllable fluid transport [92]. Similarly, hybridization of 2D materials with polymers or metal oxides enables tunable surface chemistry, adjustable porosity, and enhanced mechanical strength, achieving WCAs exceeding 155° and sliding angles below 10° [93,94]. As summarized in Table 7, WCAs in the range of 140–160° and low sliding angles can be realized through careful selection of material combinations, hierarchical structuring, and surface functionalization strategies.

The synergy of complementary properties in hybrid nanomaterials results in improved mechanical robustness, chemical stability, multifunctionality, and tunable wettability [86,87].

By integrating the complementary properties of individual components, hybrid and composite nanomaterials represent a key strategy in advanced hydrophobic surface engineering, delivering multifunctional performance that cannot be achieved with single-material systems [86,89].

The reviewed nanomaterials, including carbon–based, metal and metal oxide, polymeric, MOFs/COFs, and hybrid/composite systems, represent different approaches for engineered hydrophobicity. Carbon- and polymer–based materials offer high mechanical flexibility and chemical inertness, metal oxides provide structural strength, and MOF/COF–based frameworks contribute modular porosity and potential multifunctionality.

## 3. Mechanisms and Fabrication Methods

### 3.1. Mechanisms

The lotus leaf discussed in Section 2.1 represents a classic example of hierarchical roughness governing the Cassie–Baxter wetting state, achieving contact angles greater than 160° and sliding angles as low as 2° [95]. This hierarchical micro/nano structure is the key factor underlying their exceptional self–cleaning and water–repellent properties.

**Figure 6:**
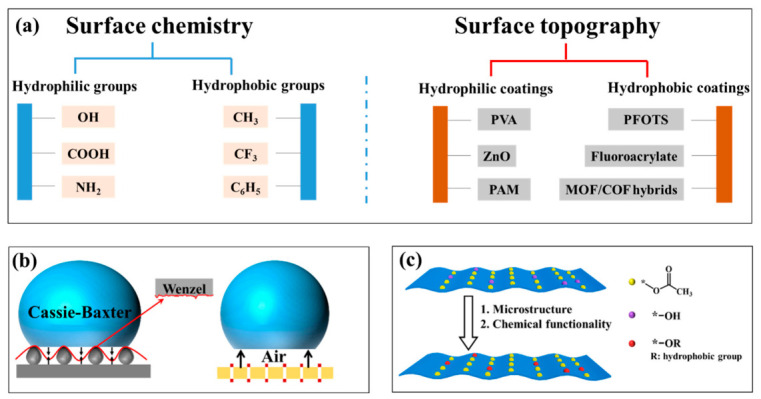
Factors in surface wettability: (**a**) functional groups affecting surface energy, (**b**) Cassie–Baxter and Wenzel wetting models, and (**c**) cellulose acetate–based smart surfaces with reversible wettability. The asterisk (*) denotes the cellulose backbone or surface attachment site. [Figure 6b,c were adapted from An et al. (2024) [98], CC BY 4.0].

Surface chemistry is also an important contributor by controlling surface free energy. Hydrophobic functional groups (e.g., –CH_3_, –CF_3_, long alkyl chains) are known to decrease interfacial energy to (<25 mJ/m_2_), whereas polar groups (–OH, –COOH) are known to increase affinity towards water via hydrogen bonding [96]. Smart surfaces take advantage of this principle by having responsive functional groups that undergo reversible chemical transformation. For example, pH– or light–sensitive coatings can be converted between superhydrophilic (<5°) and hydrophobic (>120°) states, reproducing biological adaptability [97]. Figure 6a–c emphasize the interplay between surface chemistry, topography, and functional adaptability in controlling wettability. Figure 6a shows how hydrophilic groups (–OH, –COOH, –NH_2_) enhance water affinity, while hydrophobic groups (–CH_3_, –CF_3_, aromatic rings) reduce surface free energy to below 25 mJ/m^2^, enabling water repellence [95]. Complementing this, Figure 6b illustrates Cassie–Baxter and Wenzel wetting regimes, where micro/nanostructured roughness determines whether surfaces sustain air entrapment or full liquid penetration, directly influencing superhydrophobicity or hydrophilicity [98]. Figure 6c highlights cellulose acetate (CA)–based smart surfaces that can be chemically modified with hydrophobic agents to achieve reversible wettability control. Together, these figures demonstrate that wettability design must integrate both chemistry and hierarchical roughness for optimal performance.

These effects are improved by the use of nanomaterials that allow controlled multiscale roughness and dynamic interface engineering. Engineered nanopatterns of oxides or fluoropolymers have been demonstrated to sustain superhydrophobicity even after harsh exposure to the environment [99,100,101].

The hydrophobicity in the lotus effect is inherently related to the synergy between micro- and nanoscale roughness that modifies the wetting regime by trapping air pockets and lowering the liquid–solid contact area. This dual-scale roughness destabilizes the Cassie–Baxter state, allowing water contact angles (CAs) of more than 150° and sliding angles less than 10°, typical of superhydrophobic and self–cleaning surfaces [102,103].

The hierarchical surface structures are replicated in engineered surfaces by controlling surface structures through techniques such as laser patterning, spray coating or self-assembly. Laser–induced micro/nano structuring has been demonstrated to create hybrid superhydrophobic/superhydrophilic surfaces with long–term wetting properties lasting more than 12 months [104]. Nano–roughness layers that are amphiphilic with controllable multiple scales also facilitate the dynamic wettability control switching between the stain-repellent hydrophilicity and water repellency based on the environment [105]. Such flexibility is at the core of the smart surface technologies.

Nano-textured Ti_6_Al_4_V alloy surfaces showed contact angles of 162.8° and improved corrosion resistance in comparison to smooth controls [106]. Superhydrophobic coatings produced by electrostatic powder spraying decreased ice adhesion strength by more than 80% compared to bare substrates [107], and a single–step sprayed nanostructured coating increased freezing time by several minutes at −10 °C [108].

Recent studies confirm the use of shape-memory micro/nanostructures, where the surface can be switched between superhydrophobic and hydrophilic states due to application of external stimuli, such as heat or light [109]. Furthermore, metal–polymer interfaces have previously been shown to be improved by special surfaces; roughened metal surfaces have been found to increase bonding strength by 40–60% because of improved interfacial interlocking [110]. Micro/nano surface roughness is not only essential for the hydrophobicity of lotus–inspired surfaces; it also enables multifunctionality in smart surfaces, supporting applications in self–cleaning, corrosion resistance, anti–icing, and adaptive wetting [111].

Stimuli–responsive surfaces are the next generation of smart materials that have the ability to reversibly change wettability, adhesion, and functionality under external stimulus. Such surfaces based on natural systems can change between superhydrophobic and superhydrophilic states under stimulus, such as light, temperature, pH, electric or magnetic fields, and chemical surroundings [112,113].

The reversible reorientation of chemical groups is a key mechanism for tunable wettability. For example, azobenzene-functionalized photoresponsive surfaces undergo trans–cis isomerization upon UV or visible light irradiation, triggering wettability changes from superhydrophobic (~160°) to highly hydrophilic (<20°) states [114]. Similarly, thermoresponsive polymers, such as poly(N-isopropylacrylamide) (PNIPA), collapse or expand in response to temperature, enabling controlled droplet spreading and bio-release [115]. These dynamic properties are crucial for applications in droplet manipulation, self-cleaning, and biomedical devices. Adaptive multifunctionality represents a growing area of development—switchable adhesion, for instance, is observed in surfaces with stimuli-responsive nanostructures, where a water droplet can either roll off easily or remain pinned depending on external conditions [116,117]. Contact angle hysteresis can be tuned from low (<5°) to high (>50°), facilitating precise liquid transport. Likewise, in antibacterial coatings, surfaces can alternate between killing bacteria through cationic functionalities and expelling dead cells, maintaining long-term sterility [118].

Applications include smart separation, in which the wettability of the pores of the membrane can be changed under conditions of varying pH or ionic strength, which provides selective permeability with tunable flux rates up to 500 L m^−2^ h^−1^ bar^−1^ [119]. Adaptive surfaces have also been reported to slow the formation of ice by (>300 s) under controlled conditions and increase the efficiency of water purification through controlled adsorption–desorption cycles [120,121].

Stimuli-responsive mechanisms allow surfaces to smartly conform in response to external stimuli and bridge the gap between fundamental wetting science and real-world applications, such as self–cleaning, biomedical engineering, water purification, antifouling, and energy harvesting.

A summary of these stimuli, their underlying mechanisms, and the associated quantitative change in wettability is presented in Table 8. For instance, thermoresponsive polymers, such as poly(N-isopropylacrylamide) (PNIPAM), exhibit a lower critical solution temperature (LCST) around 32 °C. Below this temperature, PNIPAM chains are extended and hydrophilic, with water contact angles below 40°, whereas above the LCST, the chains collapse, leading to hydrophobic behavior with contact angles exceeding 120° [122].

pH-responsive surfaces exploit the protonation and deprotonation of functional groups to reversibly tune wettability. For example, carboxyl-terminated nanostructures exhibit superhydrophilicity (contact angles < 10°) at acidic conditions (pH < 4) and hydrophobicity (contact angles > 130°) under basic conditions (pH > 9) due to ionization changes [123]. This tunability allows precise control of adhesion and release in biomedical and filtration systems. Similarly, photo-responsive coatings employ moieties, such as azobenzene, which undergo trans–cis isomerization upon UV irradiation, rapidly lowering contact angles from superhydrophobic (~160°) to superhydrophilic (<20°), and revert under visible light [124,125]. Such dynamic wettability is particularly useful for antifouling and antibacterial surface applications.

Magnetic-responsive systems rely on embedded nanoparticles. Hydrogels based on polyvinyl alcohol (PVA) with magnetic fillers have reversible variations in surface roughness under external magnetic fields, enabling wettability and other protein sorption to be controlled up to 200% [126]. Such coatings are used in the maritime and serve to improve the antifouling process by minimizing the adhesion forces of water by >70% when activated in the field [127]. These stimuli, thermal, pH, light, and magnetic, enable flexible control of wettability, with quantitative changes in contact angle and adhesion that are essential to applications in self-cleaning, biointerfaces, antifouling, and energy-efficient coatings.

Dynamic switching of surfaces between hydrophilic and hydrophobic states is the key to the creation of intelligent surfaces and adaptive interfaces. In contrast to fixed wettability, dynamic switching permits reversible transitions that may be induced by external stimuli, such as temperature, light, solvent composition, or chemical functionalization. Such flexibility is critical in high-technology applications in self-cleaning, water harvesting, microfluidics, and responsive membranes [128].

A fundamental mechanism is enthalpy-driven switching. A classic study demonstrated a three-state system—superhydrophobic (CA > 150°), hydrophilic (CA ~40–60°), and superhydrophilic (CA < 10°)—achieved by controlling hydrogen-bonding enthalpy at the interface [129]. This enthalpy modulation gives accurate and reversible transitions with no degradation of the surface structure. Equally, polymer brushes attached to surfaces can be tailored to respond to external cues by reorienting hydrophilic or hydrophobic side chains to adjust wettability. By adjusting polymer conformation and density, contact angles could be varied between <20° and >130° with a large range [130]. Another potential source of reversible switching lies in smart polymers that bear custom functional groups. As an example, the reaction of responsive macromolecules that contain –OH or -COOH groups changes towards hydrophilicity in a polar atmosphere, whereas fluorinated or alkylated analogues favour hydrophobicity. Such transitions are fast and reversible, allowing active regulation of droplet adhesion and spreading [131].

**Figure 7:**
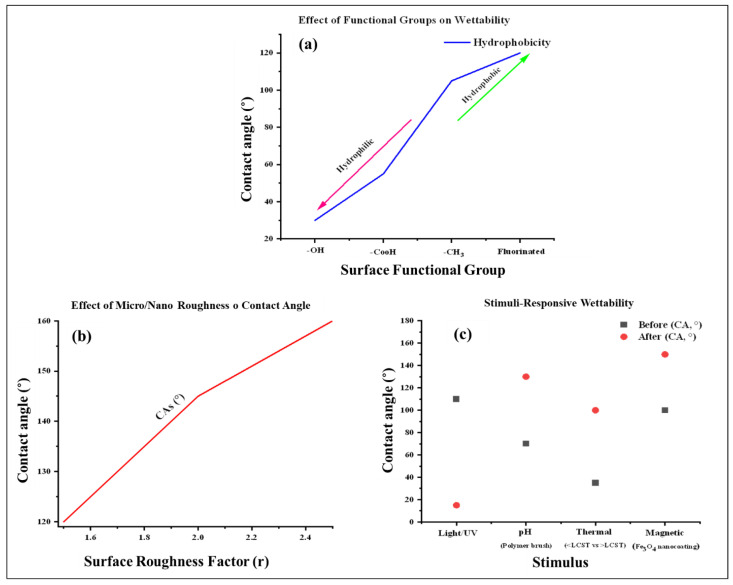
Stimuli-responsive wettability transitions between hydrophilic, hydrophobic, and superhydrophobic states, demonstrating control via surface functional groups and surface roughness.

Dynamic behavior is also shown in gas-phase switchable surfaces. Membranes have been designed by reversible amphiphilic functionalization, which allows switching between full wetting and non-wetting under varying gaseous conditions to permit selective liquid gating and transport [132]. These systems can support transfers between superhydrophilic (<5°) and superhydrophobic (>160°) surfaces with high-power fluid-control capabilities. In addition to wettability, switchable adhesion has been realized through the combination of micro/nano surface design with responsive chemistries. Surfaces may switch between high and low adhesion (contact angle hysteresis > 50° and <10°), enabling specific droplet capture and release with sensing and biomedical uses [133]. Figure 7a–c illustrate the dynamic regulation of wettability through external stimuli, where surface functional groups and nanoscale roughness act as decisive factors in tuning hydrophobic–hydrophilic transitions. Responsive functional groups (–OH, –COOH, fluorinated, or alkylated chains) reorganize under varying external cues, altering surface polarity and thereby enabling sharp shifts in contact angle [130,131]. When coupled with hierarchical roughness, these chemical changes are amplified, stabilizing extreme wetting states from superhydrophilic (<5°) to superhydrophobic (>160°) [132,134]. Such synergy ensures not only reversible transitions but also controllable adhesion [130], supporting advanced applications in water harvesting, biointerfaces, and adaptive fluid transport [128].

A combination of surface chemistry, hierarchical micro/nano roughness, and stimulus-responsive mechanisms provides a holistic strategy for developing adaptive wettability surfaces. Thermal, pH, light, and magnetic stimuli allow reversible, on-demand tuning of wettability, broadening applications in self–cleaning, biomedical interfaces, and controlled liquid handling. Dynamic switching between hydrophilic and hydrophobic modes further illustrates the potential of multistate surfaces for precise liquid adhesion and separation. Together, these strategies demonstrate that coordinated control of chemical composition, surface topography, and external stimuli is key to multifunctional smart surfaces. Challenges related to durability, response speed, and scalability remain, but the integration of these approaches represents a crucial step toward next-generation adaptive materials for practical applications.

### 3.2. Fabrication Methods

**Figure 8:**
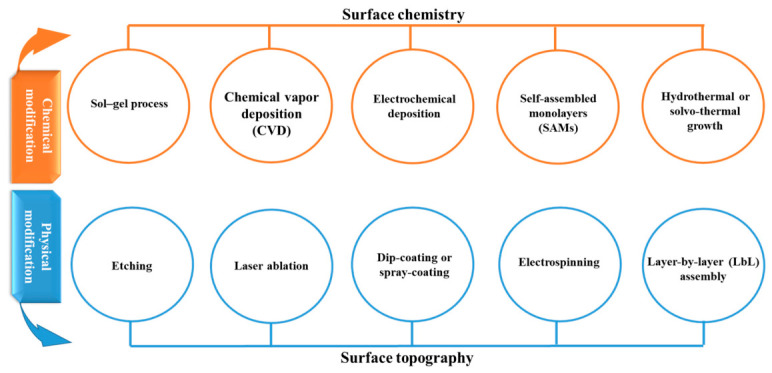
Chemical and physical modification methods for hydrophobic surface chemistry and surface topography.

The fabrication engineering of hydrophobic and superhydrophobic nanostructured surfaces demands fine control at the micro- and nanoscale of roughness and surface chemistry. Various physical, chemical and surface modification methods have been devised to form such structures with water contact angles (CAs) typically over 150° and sliding angles (SAs) lower than 10°, which are essential in self-cleaning, anti-fouling, and fluid handling applications [135,136]. These fabrication methods are presented in Figure 8, which can be categorized as physical methods, chemical synthesis routes, surface modification strategies and coating/assembly techniques, each with its own set of merits and drawbacks. Direct physical fabrication of nanostructures with tunable roughness includes etching, laser ablation and lithography. Micro/nano hierarchical structures with feature sizes ranging from 50 nm to several micrometres can be produced using dry and wet etching and greatly enhancing surface hydrophobicity [137]. These fabrication methods can be categorized into physical methods, chemical synthesis routes, surface modification strategies and coating/assembly techniques, each with its own set of merits and drawbacks.

Direct physical fabrication of nanostructures with tunable roughness includes etching, laser ablation and lithography. Micro/nano hierarchical structures with feature sizes ranging from 50 nm to several microns can be produced using dry and wet etching, and greatly enhanced surface hydrophobicity has been attained in plasma etching of silicon wafers with anisotropic micro-pillar formation.

Laser ablation provides a highly controllable method for structuring a wide range of materials. Ultrafast femtosecond laser ablation can produce periodic surface structures with feature depths of 200–500 nm, increasing CA values from ~70° (intrinsic hydrophilicity of metals) to >155° after treatment [138,139]. This method also allows the formation of hydrophilic-hydrophobic patterns to be micro-manipulated in microfluidics, with a spatial resolution of 10 µm. Nanoarrays can be designed with controlled periodicity by means of Lithography (photolithography and electron beam lithography). Photoresist-based lithography with plasma treatment has enabled hierarchical arrays with CAs greater than 160° and SAs less than 5°, which would be useful in lab-on-chip systems [135]. But lithography is expensive and time-consuming, which limits its scalability.

Low-cost production of thin hydrophobic films using alkoxysilanes of the hydrophobic type is possible by sol–gel processing. Silica films based on sol–gel are capable of CAs between 140° and 160° with a thickness of 100 to 500 nm [140]. The main parameters that affect precursor concentration, solvent ratio and ageing time are critical in determining porosity and surface energy. Chemical vapor deposition (CVD) is a general method capable of coating hydrophobic nanostructures, including fluorosilane or carbon–based coatings. Plasma-enhanced CVD has developed nanostructured surfaces with CA up to 170°, and a life over 100 abrasion cycles, and has demonstrated superiority for industrial use [136,141].

Electrospinning is used to create fibrous mats with nanofiber diameters of between 50 and 500 nm that resemble lotus-leaf surface topographies. When low–surface–energy molecules are functionalized on electrospun polymeric fibres, they deliver CAs exceeding 155° and oil–water separation efficacies of over 98% [137,142]. The high porosity (>80%) and high specific surface area (50–200 m^2^/g) of electrospun membranes are beneficial to liquid-repellent coating. Self–assembled monolayers (SAMs) provide molecular–level manipulation of surface chemistry. Fluorinated silanes or thiol SAMs can lower the surface energy to less than 20 mN/m, causing CAs to shift to more than 120° instead of the 70° with bare metal [143]. SAMs have lower mechanical performance even though they can be uniformly modified.

Polymer grafting improves adhesion and stability compared to SAMs. Grafted polymer chains, especially fluoropolymers, increase wear resistance while maintaining CAs above 150° after 10 abrasion cycles [137]. Plasma treatment modifies surfaces by both etching and deposition. Oxygen plasma can roughen polymer substrates to introduce hierarchical features, while fluorocarbon plasma deposition lowers surface energy. Combined treatments have yielded surfaces with CAs up to 165° and durability under UV irradiation for more than 500 h [141].

Spray coating is a scalable method that disperses nanoparticles or polymers onto substrates. Ultrasonic nebulization-assisted spray coating can produce uniform layers with controllable thickness (100–800 nm) and CAs up to 160° [144]. Dip coating allows uniform thin-film deposition by immersing substrates into hydrophobic sols or suspensions. Withdrawal speed and solution viscosity govern film thickness. Dip–coated silica–polymer hybrid films typically achieve CAs of 140–150° and have been demonstrated for anti-fogging applications [140]. Layer–by–layer (LbL) assembly enables sequential deposition of oppositely charged nanoparticles, polymers, or polyelectrolytes. LbL–assembled multilayers can be tuned in thickness from a few nanometers to microns with controlled porosity. When combined with fluorinated agents, these multilayers reach CAs above 155° and allow incorporation of bioactive or functional nanomaterials for multifunctional surfaces [144].

Different fabrication methods offer distinct advantages. Physical techniques provide the highest precision, chemical approaches are the most scalable and cost-effective, while surface modifications and coating strategies allow the greatest tunability and functionality. Hybrid approaches combining multiple techniques have demonstrated synergistic improvements in both hydrophobicity and mechanical robustness [136,141].

The comparison of fabrication methods highlights clear distinctions in performance, scalability, and durability. Physical techniques, such as etching, laser ablation, and lithography, yield highly precise surface structures with superior hydrophobicity, but their high cost and limited scalability restrict large-scale applications. Chemical techniques, such as sol–gel processing, CVD, and electrospinning, offer more viable routes by integrating tunable surface chemistry with porous or fibrous structures to give stable hydrophobic films and membranes that can be more widely used. Surface modification techniques, such as SAMs, grafting and plasma treatment, provide sub-micrometer control over wettability and functionality but are typically limited by durability issues during mechanical deformation or exposure to environmental factors. Meanwhile, the coating-based methods of spray coating, dip coating and layer-by-layer assembly are highly scalable and flexible, allowing the creation of multifunctional surfaces with uniform hydrophobic properties. On balance, the findings suggest that physical techniques will have the best accuracy, but chemical and coating-based approaches are the most promising in terms of efficiency, scale, and applicability in the long term in both industrial and environmental contexts.

## 4. Applications

**Figure 9:**
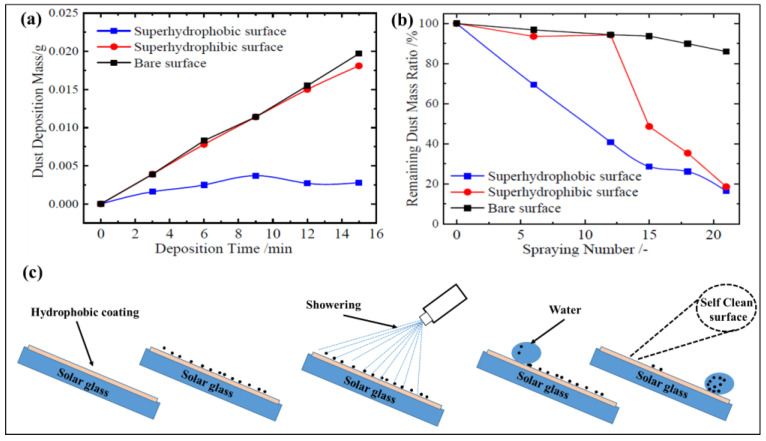
Effect of surface coatings on dust accumulation and self-cleaning of solar glass: (**a**) bare glass with significant dust deposition; (**b**) superhydrophobic coating with reduced dust accumulation; and (**c**) schematic of droplet-assisted self-cleaning process. Figure 9a,b adapted from Lu & Zheng (2022) [58], Coatings, 12(4), 502, published under CC BY 4.0.

Nanomaterials engineered for hydrophobicity have become versatile solutions to a wide range of industrial and societal challenges, offering both qualitative advantages and measurable performance improvements. Nanostructured superhydrophobic surfaces, with water contact angles exceeding 150°, enable rapid droplet roll-off, which effectively removes dirt and contaminants from windows, solar panels, and various fabrics, thereby reducing water usage and maintenance costs [145]. Figure 9a–c demonstrate the impact of these nanostructured coatings on dust accumulation and the cleaning efficiency of solar glass surfaces. Uncoated glass rapidly accumulates dust, leading to efficiency losses of up to 30% in Figure 9a, whereas superhydrophobic coatings significantly reduce deposition by more than 60% compared to bare glass in Figure 9b. The cleaning mechanism, illustrated in Figure 9c, shows rolling droplets removing particulates and contaminants, confirming the Cassie–Baxter wetting regime characteristic of superhydrophobic surfaces. Beyond solar panels, these coatings also reduce bacterial adhesion by over 80% on medical and marine surfaces, extending service life while minimizing the need for toxic biocides [144,146].

Anti-icing is another important application of hydrophobic nanocoatings, which can reduce ice adhesion strength by up to 90%. This property is critical for aerospace components, wind turbine blades, and other systems operating in cold environments. Superhydrophobic coatings also enhance corrosion resistance, improving metal durability and reducing corrosion rates by over 60% [147,148]. Water repellency based on engineered hydrophobic nanomaterials and self-cleaning surfaces exploits the ability of water repellency and photocatalytic effects to lower the amount of dirt on a surface to enhance efficiency and lessen maintenance. Notably, the solar panels that have been hydrophobized have demonstrated up to 30% greater power output than uncoated panels since dust deposition is reduced [149]. Recent studies reported that functionalized cellulose-based textiles maintained over 90% self–cleaning efficiency after 50 washing cycles [150].

Hydrophobic nanostructured surfaces reduce bacterial adhesion by over 80%, while marine coatings incorporating nanocomposites can extend service life by 3–5 years compared to conventional paints [151,152]. Engineered hydrophobic and icephobic nanostructures have shown significant promise by reducing ice adhesion strength by 70–90%, allowing passive de-icing under minimal shear forces [153]. For example, superhydrophobic aircraft coatings reduced ice accumulation by up to 80% in laboratory simulations. Similarly, wind turbine blades treated with nanostructured coatings demonstrated a 25% increase in energy generation in cold climates [154]. Oil–water separation remains a critical environmental challenge, especially in industrial wastewater treatment and marine oil spill remediation [155]. Superhydrophobic/superoleophilic nanomaterials have enabled membranes and sponges with separation efficiencies of >98%, even under harsh pH and salinity conditions [156].

Current limitations involve durability and fouling resistance. Prolonged exposure to viscous oils, organic solvents, and environmental contaminants often reduces performance, while regeneration of materials can be costly or inefficient. Furthermore, many nanocoatings still rely on fluorinated compounds, raising environmental concerns. Future developments are focusing on biodegradable and recyclable nanomaterials, such as cellulose-based composites and graphene-derived structures, which maintain high efficiency while reducing ecological risks. Integrating self-healing functionalities to restore hydrophobicity after prolonged use is another promising direction. With continuous advancements, next-generation oil–water separation materials could achieve long-term efficiency above 95% for over 100 cycles of reuse, making them viable for large-scale wastewater treatment and emergency oil spill recovery.

Hydrophobic nanomaterials provide a sustainable strategy for corrosion resistance by serving as both physical and chemical barriers between metals and corrosive environments. Recent studies have demonstrated that superhydrophobic coatings can reduce corrosion current densities by over two orders of magnitude, thereby extending the service life of alloys and steel under saline conditions [157].

The long-term durability of hydrophobic coatings under mechanical abrasion, ultraviolet radiation, chemical exposure, and harsh environmental conditions remains insufficient for many practical applications. Although bio-based materials, such as cellulose nanofibers, lignin, and other renewable resources, have emerged as promising environmentally friendly alternatives, their performance and durability often remain inferior to those of conventional fluorinated systems. Furthermore, advanced fabrication techniques, including lithography, chemical vapor deposition, and laser texturing, may face scalability and economic limitations that hinder industrial adoption. Table 9 summarizes the challenges of sustainability, environmental considerations, and future development directions associated with current hydrophobic and superhydrophobic surface technologies.

In underwater vehicles, biomimetic shark-skin-inspired coatings achieved drag reductions of up to 25%, enhancing fuel efficiency and manoeuvrability [158]. Similarly, microfluidic devices benefit from SHS, where flow resistance decreases by approximately 40%, enabling faster and more efficient chemical and biological analyses [159]. Hydrophobic nanomaterials have transformed wearable and intelligent textiles by providing water repellency, breathability and multifunctionality. The superhydrophobic textiles that have water contact angles of more than 155° repel stains and liquids and are comfortable to wear. Recent advances in fluorine-free finish obtained over 90% water resistance in 50 wash cycles, making them last longer in daily use [160]. Nanostructured surfaces also allow breathable and protective fabrics, where the air permeability is more than 200 mm/s, yet still the fabrics do not allow water to penetrate them. The uses of wearable applications are not confined to clothing and smart textiles that use wearable sensors, energy harvesters, and antimicrobial agents to track health status and protect individuals [161].

Future opportunities lie in the development of multifunctional, environmentally friendly nanocomposites that combine hydrophobicity with additional features, such as photocatalysis, antimicrobial activity, photothermal de-icing, or self-healing. Bio-inspired designs, including lotus leaves, shark skin, and penguin feather-like structures, hold promises for creating sustainable antifouling, drag-reducing, and ice-phobic surfaces. Durability and scalability can be further enhanced through advanced fabrication strategies, such as micro/nano-manufacturing and additive manufacturing. Optimized coatings are projected to achieve nearly 100% ice release, over 90% corrosion resistance after 1000 h, and 95% textile water repellency after 100 wash cycles. Collectively, these developments will enable safer, more energy-efficient, and sustainable technologies, underscoring the critical role of hydrophobic nanomaterials in next-generation applications.

## 5. Conclusions

Nanotechnology has dramatically transformed the field of hydrophobic surface engineering, enabling the creation of surfaces with tunable wettability and multifunctional properties. The transition from natural models, such as the lotus leaf effect, to engineered surfaces represents a significant evolution based on a deep understanding of surface topography and chemistry. Early biological models, featuring micro- and nanoscale hierarchical structures of lotus leaves, provided a blueprint for controlling wetting behavior through dual-scale roughness and low-energy surface chemistry. Building on these concepts, advanced fabrication techniques, including laser ablation, lithography, etching, and self-assembly, allow precise control over surface topography and chemical composition, yielding superhydrophobic and stimuli-responsive surfaces with tailored functionality.

A wide variety of materials are now employed, including fluorinated polymers, PDMS, metal-oxide nanostructures, MOF/COF frameworks, and hybrid nanocomposites that synergistically combine the advantages of each component. The key mechanisms underlying these properties involve hierarchical roughness that traps air pockets to sustain the Cassie–Baxter state, as well as dynamic chemical functionalities that enable reversible wettability and multifunctionality. Stimuli-responsive surfaces further extend capabilities by responding to pH, light, or temperature, allowing reversible switching between hydrophilic and hydrophobic states.

Hydrophobic nanomaterials are poised to play a central role in future technological innovations. Critical challenges remain, particularly in ensuring durability under harsh conditions, scalability, and environmental safety, especially concerning fluorinated and metallic components. Future research is directed toward environmentally friendly, multifunctional nanocomposites and bioinspired designs that integrate antimicrobial, self-healing, and energy-harvesting functions. Stimuli-responsive, multistate surfaces that dynamically alternate between hydrophilic and hydrophobic states may replicate the adaptability observed in nature, enabling transformative applications in biomedical devices, energy-efficient systems, marine technologies, and sustainable materials. Overcoming current limitations in long-term stability, fabrication scalability, and environmental compatibility will be essential for realizing the full potential of these intelligent hydrophobic surfaces.

## Figures and Tables

**Table 1:** Physicochemical and structural parameters influencing hydrophobic and superhydrophobic behavior.

No	Parameter	Description	Effect on Hydrophobicity	Materials	Refs.
1	Surface Energy	Energy associated with solid–liquid and solid–vapor interfaces	Low surface energy materials repel water; high surface energy materials attract water	Fluoropolymers: ~10–20 mN/m (hydrophobic, θ ≈ 110°); Metals: ~500–2000 mN/m (hydrophilic, θ < 60°)	[23,24,28]
2	Surface Chemistry	Presence of functional groups such as –CH_3_, –CF_3_, –Si–O–	Hydrophobic groups lower γ_SL, improving CA stability; hydrophilic groups increase wettability	Fluorosilane coatings increased CA from 75° (bare Si) to >115°	[28,31]
3	Surface Roughness	Geometric irregularities at micro- or nanoscale	Amplifies intrinsic wettability (Wenzel); stabilizes Cassie state when combined with low-energy chemistry	Roughened glass beads showed higher flotation performance due to CA increase; θ rose from ~65° (smooth) to >95° (roughened)	[28,29]
4	Surface Morphology	Topography and texture influencing the wetting state	Structured morphologies (pillars, grooves) reduce liquid–solid contact, increasing hydrophobicity	Er_2_O_3_ thin films maintained CA > 120° due to morphology-driven hydrophobicity	[30,31]
5	Hierarchical Structures	Combined micro- and nanoscale features	Stabilize Cassie–Baxter state; enhance robustness against wear and contamination	Lotus leaf: θ = 160–170°, slide angle 2–5°; Plasma nanotexturing: θ > 160° with anti-reflective function	[25,32,33]
6	State Transition (Wenzel-Cassie)	Droplet states depending on roughness and energy	Cassie state, high CA & low hysteresis; Wenzel state, pinned droplets with higher hysteresis	Sliding angle <10° in Cassie regime vs. >30° in Wenzel regime	[25,26,27]

**Table 2:** The biomimetric translation of natural hydrophobic models, surface morphologies, wetting behaviors, and technological applications.

No	Model	Surface Chemistry	Wetting	Applications	Ref.
1	Lotus Leaf	Micro-papillae (10–20 μm) + nanoscale wax crystals (~100 nm); low surface energy (20–25 mN/m)	Superhydrophobicity: contact angle > 160°, sliding angle < 10°; self-cleaning effect	Self–cleaning coatings, anti-fouling paints, corrosion protection	[34,35,38]
2	Rose Petal	Micro–papillae with nano-cuticular folds	High contact angle (>150°) but high adhesion (droplet pinning)	Droplet immobilization in microfluidics, biochemical assays	[39,40]
3	Butterfly Wing	Overlapping scales with hierarchical roughness	Anisotropic hydrophobicity: directional droplet rolling	Water guiding surfaces, optical–wetting integrated materials	[39,40]
4	Water Strider Leg	Dense superhydrophobic hairs (~50 μm long)	Supports insects on water via surface tension; floating stability	Anti-wetting textiles, water-repellent outdoor gear, buoyant devices	[39,40]
5	Artificial Lotus-Inspired Surfaces	Engineered hierarchical micro–nano textures + hydrophobic coatings	Replicate lotus repellency: contact angle >155–160°, robust self-cleaning	Infrastructure protection, cultural heritage conservation, biomedical devices	[34,41,42]

**Table 3:** Hydrophobic and superhydrophobic characteristics of carbon–based nanomaterials.

No	Nanomaterial	Intrinsic WCA	Modified WCA	Strategy	Applications	Ref.
1	Carbon Nanotubes (CNTs)	~95–110°	>165°	Fluorinated polyurethane matrix, silica hybridization	Durable superhydrophobic coatings, abrasion resistance	[48,49]
2	Graphene	~90–95°	150–160°	Fluorination, alkyl–silane grafting	Corrosion–resistant surfaces, self–cleaning	[46,50]
3	Graphene Oxide (GO)	~70–80°	150–155°	Fluorosilane functionalization, surface roughening	Oil–water separation, self–cleaning	[44,45,51]

**Table 4:** Hydrophobic performance of metal and metal oxide nanomaterials.

No	Nanomaterial	Intrinsic WCA	Modified WCA	Modification	Applications	Ref.
1	SiO_2_	60–80°	155–160°	Fluorosilane functionalization, electrospinning, polymer composites	Self-cleaning surfaces, oil–water separation, anti-fouling coatings	[62,63]
2	TiO_2_	50–75°	150–155°	Fluorosilane grafting, low-surface-energy functionalization	Photocatalytic self-cleaning, anti-fouling, environmental remediation	[64,65]
3	ZnO	45–70°	150–155°	Fluorosilanes, perfluoroalkyl chains, polymer matrix incorporation	Anti-corrosion coatings, UV-resistant surfaces, oil–water separation	[56,64]
4	Al_2_O_3_	55–80°	150–155°	Alkyl-silane functionalization, polymer composites	Barrier coatings, corrosion resistance, mechanical reinforcement	[55]
5	Fe_3_O_4_	50–75°	150–155°	Fluorosilane or alkyl-silane functionalization	Magnetic oil–water separation, environmental cleanup, multifunctional coatings	[66]

**Table 5:** Functional perspective on polymeric nanomaterials for engineered hydrophobicity.

No	Polymer Type	Functionalization	Achieved WCA	Advantages	Applications	Ref.
1	Fluorinated Polymers	Hierarchical micro/nanostructuring, low-surface-energy fluorination	120–160°	Extremely low surface energy, chemical stability, transparency	Self-cleaning surfaces, anti-fouling coatings, water–oil separation	[69,71]
2	PDMS	Crosslinking, nanoparticle incorporation, surface texturing	110–150°	Flexibility, chemical/thermal stability, tunable elasticity	Medical devices, flexible coatings, microfluidics	[70,75]
3	Teflon-like Coatings	High fluorine content, nanostructured surfaces	150–160°	Robust water repellency, mechanical durability	Industrial pipelines, protective coatings, anti-adhesion surfaces	[68]
4	Polymer Nanocomposites	Polymer matrices reinforced with SiO_2_, TiO_2_, ZnO nanoparticles	140–160°	Enhanced surface roughness, mechanical strength, multifunctionality	Anti-corrosion coatings, superhydrophobic membranes, self-cleaning films	[72,73,74]

**Table 6:** A summary of MOFs and COFs for engineered hydrophobic surfaces.

No	Framework	Structure	Functionalization	Achieved WCA	Advantages	Applications	Ref.
1	MOFs (e.g., HKUST-1, ZIFs)	Nanoporous 3D frameworks	Fluorination, alkylation, ligand functionalization	150–155°	High surface area, selective adsorption, tunable chemistry	Oil–water separation, water-repellent coatings, adsorption-based devices	[78,79]
2	COFs (e.g., fluorinated COFs, 2D/3D networks)	Highly ordered porous covalent network	Fluorination, alkylation, hybridization	155–160°	Chemical/thermal stability, tunable pore chemistry, low solid–liquid contact	Hydrophobic coatings on textiles, chemical-resistant films, filtration membranes	[77,80]
3	MOF/COF Hybrids	MOF core + COF shell	MOF functionalization + COF surface fluorination	155–160°	Combines selective adsorption, structural stability, and superhydrophobicity	Advanced water–oil separation, multifunctional sensors, protective coatings	[79,84]

**Table 7:** A summary of hybrid and composite nanomaterials for engineered hydrophobicity.

No	Composite	Components	Strategy	Achieved WCA	Advantages	Applications	Ref.
1	Polymer–Nanoparticle Composites	Polymers + SiO_2_, TiO_2_, ZnO nanoparticles	Hierarchical structuring, surface functionalization	140–160°	Enhanced surface roughness, mechanical durability, chemical stability	Self-cleaning coatings, anti-corrosion films, water–oil separation	[88,89]
2	Carbon-Based Hybrids	CNTs, graphene, graphene oxide + polymers or metal oxides	Nanocomposite integration, fluorination, surface texturing	150–160°	Multifunctionality, mechanical reinforcement, tunable wettability	Anti-fouling coatings, microfluidics, superhydrophobic films	[86,87]
3	MOF/COF Hybrids	MOFs@COFs + polymers or carbon materials	Core–shell design, surface functionalization	155–160°	High porosity, selective adsorption, chemical stability	Oil–water separation, filtration membranes, sensors	[93,94]
4	Magnetic Nanocomposites	Fe_3_O_4_ + polymers/silica	Surface functionalization, hierarchical architecture	150–155°	Magnetic response + superhydrophobicity, reusability	Environmental remediation, controllable fluid transport	[91,92]

**Table 8:** A comparison of thermal, pH, light, and magnetic stimuli-responsive wettability mechanisms, highlighting contact angle transitions, and applications.

No	Stimulus	Mechanism	Wettability	Features	Applications	Ref.
1	Thermal (PNIPAM, LCST ~32 °C)	Coil-globule transition of polymer chains alters hydrophilicity	<40° (hydrophilic, below LCST) >120° (hydrophobic, above LCST)	Sharp switch in droplet adhesion; tunable reversible transitions	Drug delivery, smart coatings, and biomedical devices	[122]
2	pH (carboxylated nanostructures)	Protonation/deprotonation of –COOH groups	<10° (superhydrophilic, pH < 4) >130° (hydrophobic, pH > 9)	pH-controlled droplet spreading and detachment	Filtration, controlled adhesion/release, responsive membranes	[123]
3	Light (azobenzene, UV/Vis)	Trans–cis photoisomerization changes surface polarity	~160° (superhydrophobic, dark) <20° (superhydrophilic, UV)	Rapid transition within seconds; reversible with visible light	Antifouling, antibacterial switching, microfluidics	[124,125]
4	Magnetic (PVA hydrogels with nanoparticles)	Magnetic field induces surface roughness and reorientation	Variable CA shifts; sorption capacity enhanced by ~200% under field	Water adhesion forces reduced by >70% with magnetic activation	Protein sorption, antifouling in maritime coatings, and biomedical interfaces	[126,127]

**Table 9:** Sustainability assessment of hydrophobic and superhydrophobic surface technologies, highlighting current advantages, limitations, and future research directions.

No	Sustainability Aspect	Current Status	Limitation	Future Direction	Ref.
1	Fluorinated coatings	Excellent water repellency and low surface energy	Environmental persistence, potential ecological concerns	Development of fluorine-free hydrophobic materials	[3,14]
2	Bio-based materials (cellulose, lignin)	Renewable and environmentally friendly	Lower durability and long-term stability	Hybrid bio-based nanocomposites and surface reinforcement	[12,14]
3	Nanostructured coatings	High WCA (>150°) and multifunctionality	Mechanical abrasion and surface degradation	Self-healing and wear-resistant architectures	[74,75]
4	Lithography and laser texturing	Precise hierarchical structuring	High manufacturing cost and limited scalability	Cost-effective large-area fabrication methods	[11]
5	CVD and plasma processes	Excellent coating uniformity and performance	Energy-intensive processing	Low-energy and scalable deposition techniques	[8,11]
6	MOF/COF-based systems	High porosity and multifunctionality	Complex synthesis and industrial scalability challenges	Simplified synthesis and large-scale production	[83]
7	Hybrid nanocomposites	Enhanced durability and multifunctionality	Material complexity and recycling concerns	Sustainable multifunctional hybrid systems	[94]
8	Smart hydrophobic surfaces	Adaptive and stimuli-responsive performance	Limited long-term reliability and high fabrication complexity	Robust, scalable, and environmentally benign smart coatings	[97]

## Data Availability

No new data were created or analyzed in this study. Data sharing does not apply to this article.
